# Multidrug Resistance-Associated Proteins 3 and 5 Play a Role in the Hepatic Transport of Mercuric Conjugates of Glutathione

**DOI:** 10.3390/ijms26031194

**Published:** 2025-01-30

**Authors:** Maria Eduarda Andrade Galiciolli, Lucy Joshee, Cláudia S. Oliveira, Jennifer L. Barkin, Christy C. Bridges

**Affiliations:** 1 Department of Biomedical Sciences, Mercer University School of Medicine, Macon, GA 31207, USA; dudagaliciolli@hotmail.com (M.E.A.G.); joshee_l@mercer.edu (L.J.); 2 Instituto de Pesquisa Pelé Pequeno Príncipe, Curitiba 80250-060, PR, Brazil; claudia.bioquimica@yahoo.com.br; 3 Faculdades Pequeno Príncipe, Curitiba 80230-020, PR, Brazil; 4 Department of Community Medicine, Mercer University School of Medicine, Macon, GA 31207, USA; barkin_jl@mercer.edu

**Keywords:** mercury, multidrug resistance-associated transporter, liver, transport, heavy metals

## Abstract

Multidrug resistance proteins (MRPs) are transporters for metabolic waste and xenobiotics and are known to export a wide range of substances from renal tubular cells. This study aimed to define and characterize the transport of mercuric conjugates of glutathione (GSH-Hg-GSH) in inside-out membrane vesicles containing MRP3 and MPR5. The functionality of the MRP3 and MRP5 vesicles was confirmed by measuring the uptake of [^3^H]-estradiol and 5-6-carboxy-2′,7′-dichloro-fluorescein (CDCF) over time (at 1, 5, 15, and 30 min). The uptake of GSH-Hg-GSH, containing radioactive mercury ([^203^Hg]), was measured in each set of membrane vesicles over time, and the findings suggest that GSH-Hg-GSH is a substrate of MRP3 and MRP5. The saturation kinetics were also analyzed by measuring the uptake of 10 µM GSH-[^203^Hg]-GSH in the presence of 25, 50, or 100 µM unlabeled GSH-Hg-GSH for 5 min at 37 °C. The transport of GSH-Hg-GSH by MRP3 (*V_max_* = 25.6 µM; *K_m_* = 2.8 µM) and MRP5 (*V_max_* = 32.9 µM; *K_m_* = 4.9 µM) was saturable. These findings are the first to show that MRP3 and MRP5 are capable of mediating the export of any form of mercury.

## 1. Introduction

Mercury (Hg) is a toxic heavy metal known for its harmful effects on human health. Its toxicity is associated with the inhibition of sulfhydryl enzymes, generation of reactive oxygen species (ROS), and depletion of thiols and selenols, among other effects [1]. Hg has been directly linked to deleterious effects on the central nervous system, including neurotransmitter dysregulation, synaptic dysfunction, mitochondrial impairment, DNA damage, and autophagic dysregulation. These effects may contribute to the development of neurodegenerative and autoimmune diseases, including Parkinson’s disease and Alzheimer’s disease [2,3,4]. Hg exists in the environment as elemental (Hg^0^), inorganic (Hg^2+^), and organic forms (e.g., CH_3_Hg^+^). All forms of Hg are toxic to humans, but the toxicity depends on multiple factors, such as the duration and route of exposure and the chemical form to which one is exposed [5,6].

Humans may be exposed to Hg through oral (ingestion of contaminated fish), dermal (use of Hg-containing skin creams), and respiratory routes (inhalation of Hg vapor), with the majority of exposure occurring through the ingestion of contaminated fish. Although humans may be exposed to elemental or organic forms of Hg, these forms are converted to inorganic forms within the blood and tissues after inhalation/ingestion [7,8,9]. After oral exposure to inorganic Hg, more Hg accumulates in the kidney compared to other organs [10]. Similarly, a study with mice injected with thimerosal (ethylmercury) also showed that the highest levels of Hg were found in the kidneys [11]. These findings show that the kidney is an important target for mercuric ions [12].

Mercuric ions have a high affinity for sulfhydryl groups; thus, they form strong bonds with free sulfhydryl (thiol) groups located on endogenous biomolecules such as albumin, glutathione, and cysteine [13,14]. Most of the mercuric ions in blood are bound to sulfhydryl groups on proteins like albumin, forming Hg–albumin complexes [15,16]. Previous in vivo studies provided evidence that the liver plays an important role in the urinary excretion of circulating mercuric ions [17,18]. It was suggested that Hg–albumin complexes in the blood are transported to the liver where they are taken up at the sinusoidal membrane of hepatocytes and processed intracellularly to remove the mercuric ion from the albumin molecule [17]. The mercuric ions then bind to intracellular glutathione (GSH), creating mercuric conjugates of GSH (GSH-Hg-GSH). GSH-Hg-GSH conjugates have been shown to be exported from hepatocytes at the canalicular membrane via the multidrug resistance-associated protein (MRP) 2 [19,20]. Yet, it also appears that these conjugates may also be exported to the sinusoidal membrane. This process would deliver the conjugates to the blood for subsequent glomerular filtration and urinary excretion.

The mechanisms that mediate the export of GSH-Hg-GSH at the sinusoidal membrane of hepatocytes have not been identified; however, it is reasonable to postulate that MRPs may participate in this process. The MRP family is a large group of membrane transporters that mediate the export of numerous metabolic wastes and xenobiotics [20]. MRP3 plays a key role in bile salt physiology, drug metabolism, and detoxification by balancing drug–glucuronide transport with MRP2 [21], while MRP5 has been shown to transport organic anions and GSH conjugates (Supplementary Figure S1) [22]. Both transporters actively transport substrates across membranes, with conformational changes induced by ATP binding and hydrolysis [23,24].

As noted above, MRP2 has been shown to mediate the export of mercuric conjugates at the canalicular membrane of hepatocytes [19,20]. MRP2 is localized exclusively in the canalicular membrane; thus, it does not play a role in the sinusoidal transport of Hg. However, MRP3 and MRP5 are localized in the sinusoidal membrane [25]; therefore, we hypothesize that these transporters are involved in the export of mercuric ions from within hepatocytes into the blood. There are no published studies indicating that MRP3 and/or MRP5 can mediate the transport of any species of Hg; therefore, this represents an important knowledge gap. Specifically, we hypothesize that MRP3 and MRP5 facilitate the sinusoidal export of GSH-Hg-GSH, enabling its transport from the liver to the bloodstream and ultimately promoting renal excretion. This is the first study designed to evaluate and characterize the ability of MRP3 and MRP5 transporters to mediate the transport of any species of Hg.

## 2. Results

### 2.1. Analyses of Hg Transport into Membrane Vesicles Containing MRP3

**Figure 1:**
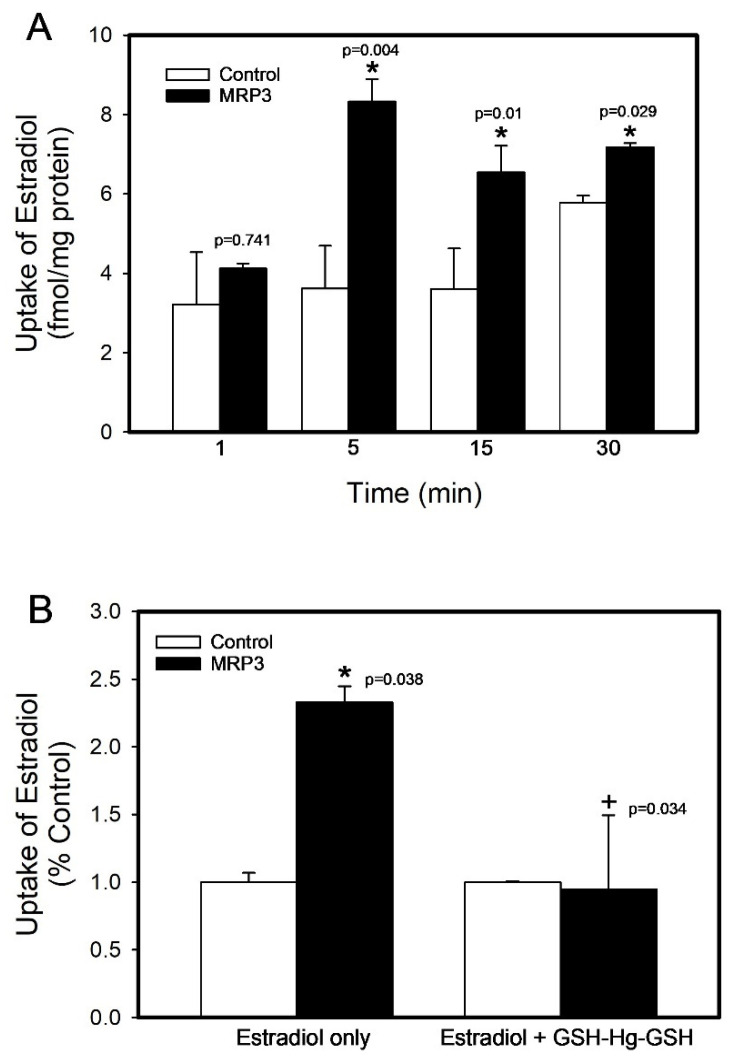
The viability of inside-out membrane vesicles containing MRP3 was assessed by measuring (**A**) the uptake of 100 nM [^3^H]-estradiol over time and (**B**) in the presence or absence of unlabeled GSH-Hg-GSH (500 µM). The results were analyzed using a two-way ANOVA, followed by Tukey’s post hoc test, and are presented as the mean ± the standard error, n = 3. * Significantly different (*p* < 0.05) from the corresponding control vesicles. + Significantly different (*p* < 0.05) from the MRP3 vesicles exposed to estradiol only.

The viability of inside-out membrane vesicles was assessed by measuring the uptake of [^3^H]-estradiol to validate the activity of MRP3. The time course of the estradiol uptake (1, 5, 15, and 30 min) is shown in Figure 1A. The results revealed a significant increase in the estradiol uptake by MRP3 in membrane vesicles at 5 (130.20%), 15 (81.60%), and 30 (24.06%) minutes compared to the control group (*p* < 0.05). There were no significant differences in the uptake among the control vesicles. In a separate experiment, the uptake of [^3^H]-estradiol was measured in control or MRP3 vesicles in the presence or absence of unlabeled GSH-Hg-GSH (Figure 1B). These data showed a significant increase in the estradiol uptake into MRP3 vesicles after 5 min (133%) compared with the control group (*p* < 0.05). In the presence of GSH-Hg-GSH, the uptake of [^3^H]-estradiol into MRP3 vesicles was significantly decreased (59.29%; *p* < 0.05) and was not different than that of the control vesicles.

**Figure 2:**
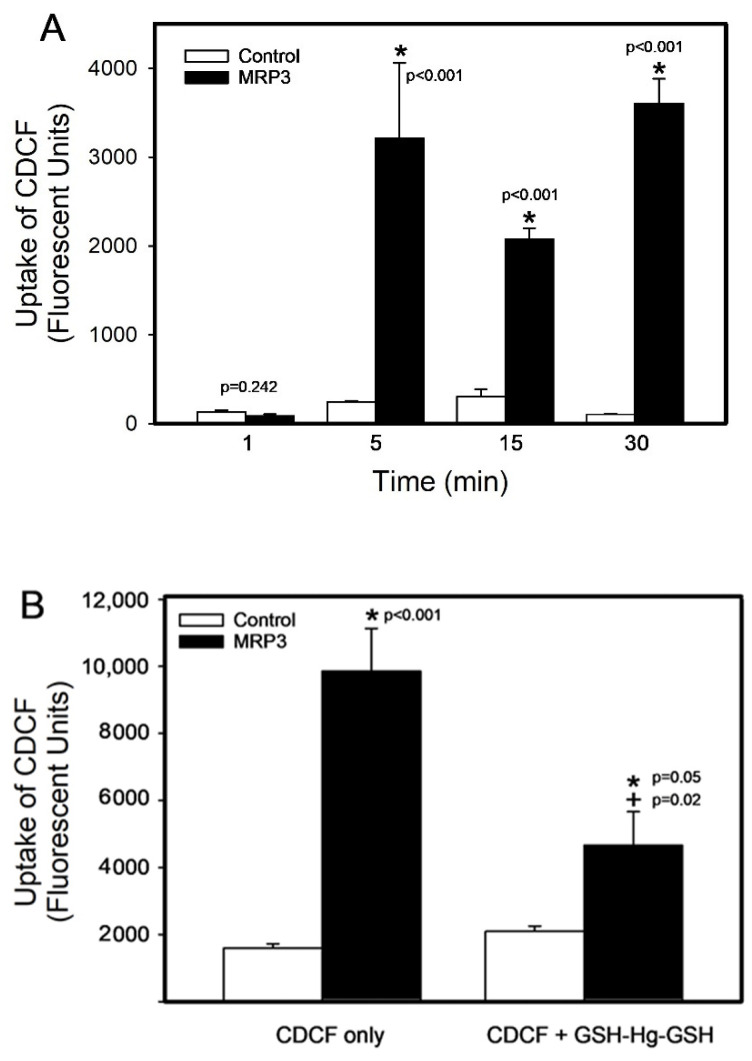
The uptake of CDCF into control or MRP3 inside-out membrane vesicles. The uptake of CDCF (**A**) was measured over time (at 1, 5, 15, and 30 min) and (**B**) in the presence or absence of GSH-Hg-GSH. The results were analyzed using a two-way ANOVA, followed by Tukey’s post hoc test, and are presented as the mean ± the standard error, n = 3. * Significantly different (*p* < 0.05) from the mean of the corresponding control vesicles. + Significantly different (*p* < 0.05) from the mean of MRP3 vesicles exposed to CDCF only.

The uptake of CDCF, a known substrate of MRP3, was measured over time (at 1, 5, 15, and 30 min) to serve as another measure of validation (Figure 2A). The uptake of CDCF was significantly greater in MRP3 vesicles than in control vesicles after 5 (1220%), 15 (587.3%), and 30 (3377%) minutes of uptake. There were no significant differences in the uptake among the different groups of control vesicles. The uptake of CDCF in the presence or absence of GSH-Hg-GSH is shown in Figure 2B. The addition of 500 µM unlabeled GSH-Hg-GSH significantly reduced (122.56%) the uptake of CDCF into MRP3 vesicles (*p* < 0.05).

**Figure 3:**
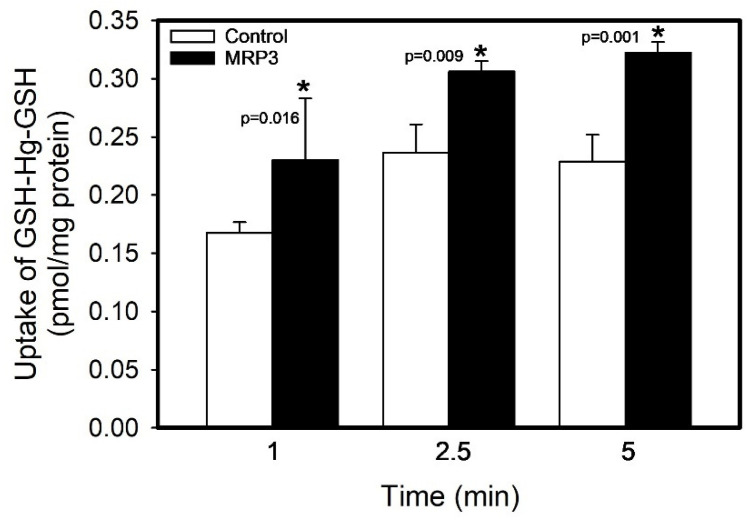
Time course of the GSH-Hg-GSH uptake by MRP3. The results were analyzed using a two-way ANOVA, followed by Tukey’s post hoc test, and are presented as the mean ± the standard error, n = 3. * Significantly different from the mean of the corresponding control vesicles.

To directly assess the ability of MRP3 to mediate the transport of GSH-Hg-GSH, the uptake of GSH-[^203^Hg]-GSH was measured over time (at 1, 2.5, and 5 min) at 37 °C (Figure 3). The uptake of GSH-[^203^Hg]-GSH in MRP3 vesicles after 1 (37.37%), 2.5 (29.45%), and 5 (40.96%) min was significantly greater than that in corresponding control vesicles (*p* < 0.05). There were no significant differences in the uptake among the different groups of control vesicles.

**Figure 4:**
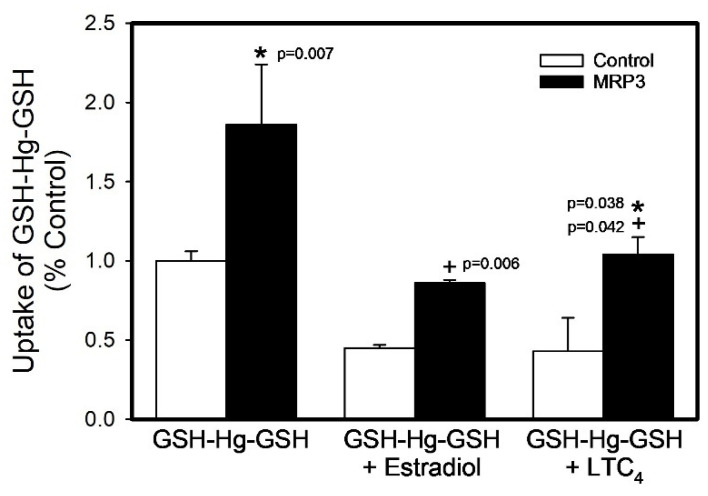
The inhibition of GSH-Hg-GSH by estradiol or leukotriene C_4_ (LTC_4_). The uptake of GSH-Hg-GSH by MRP3 was measured in the presence of known substrates of MRP3, estradiol or LTC_4_. The results were analyzed using a two-way ANOVA, followed by Tukey’s post hoc test, and presented as the mean ± the standard error, n = 3. * Significantly different (*p* < 0.05) from the mean of the corresponding control vesicles. + Significantly different (*p* < 0.05) from the mean of the corresponding vesicles exposed to GSH-Hg-GSH in the absence of estradiol or LTC_4_.

The uptake of GSH-[^203^Hg]-GSH in the presence and absence of estradiol or leukotriene C_4_ (LTC_4_), two known substrates of MRP3, is shown in Figure 4. The addition of estradiol and LTC_4_ competed with GSH-Hg-GSH at the site of MRP3 and resulted in a significant decrease (53.06% and 43.1%, respectively) in the GSH-Hg-GSH uptake when compared to that of MRP3 vesicles in the absence of those compounds (*p* < 0.05).

**Figure 5:**
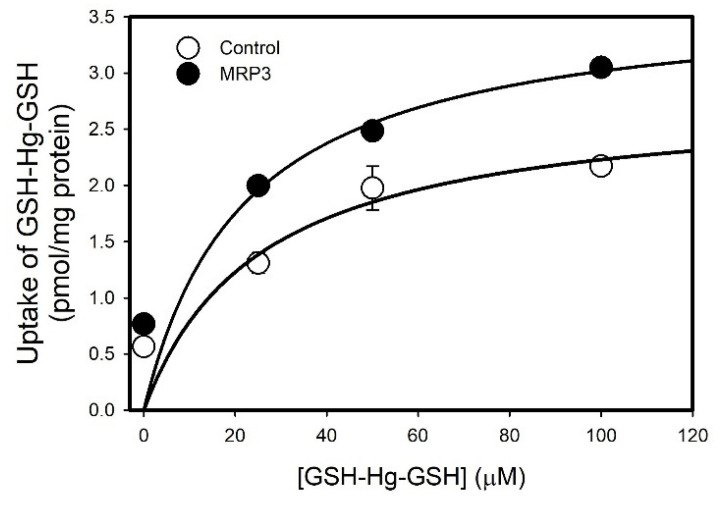
Michaelis–Menten kinetics of GSH-Hg-GSH transport. Transport kinetics of GSH-Hg-GSH uptake by MRP3 were assessed in inside-out membrane vesicles. Results are presented as mean ± standard error, n = 3.

The Michaelis–Menten kinetics of GSH-Hg-GSH transport by MRP3 were analyzed by measuring the uptake of GSH-[^203^Hg]-GSH in the presence of unlabeled GSH-Hg-GSH. The maximum velocity (*V_max_*) was estimated to be 25.57 pmol mg protein^−1^ min^−1^, and the *K_m_* was calculated to be 2.8 μM for MRP3 vesicles. For control vesicles, the *V_max_* was estimated to be 21.97 pmol mg protein^−1^ min^−1^, and the *K_m_* was 3.68 μM (Figure 5)

### 2.2. Analysis of GSH-Hg-GSH Transport into Membrane Vesicles Containing MRP5

**Figure 6:**
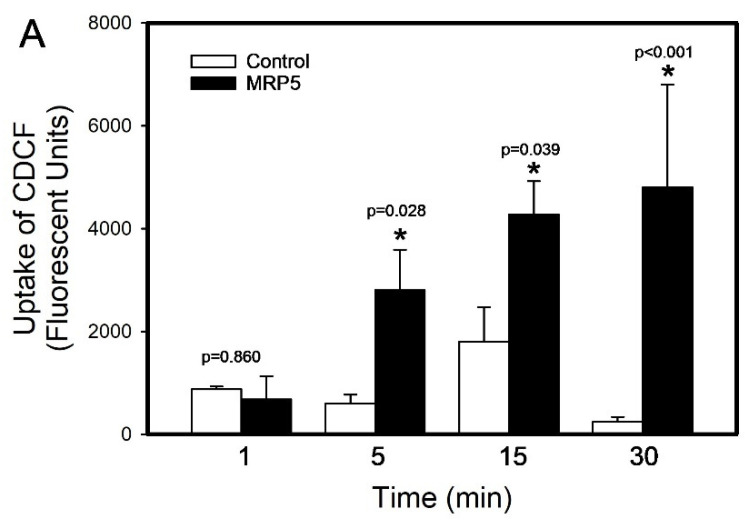

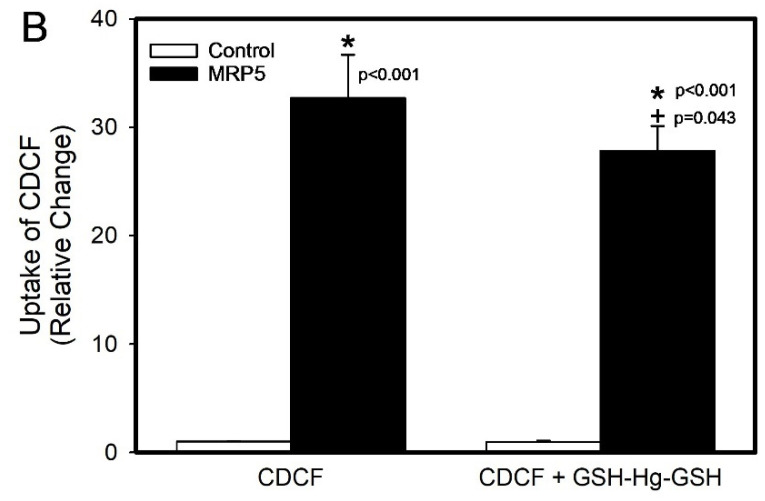
The viability of inside-out membrane vesicles containing MRP5 was assessed by measuring the uptake of CDCF over time (at 1, 5, 15, and 30 min (**A**)) and (**B**) the CDCF uptake in the presence or absence of GSH-Hg-GSH. The results were analyzed using a two-way ANOVA followed by Tukey’s post hoc test and are presented as the mean ± the standard error, n = 3. * Significantly different (*p* < 0.05) from the mean of the control vesicles. +, significantly different (*p* < 0.05) from the MRP5 vesicles exposed to CDCF only.

The viability of inside-out membrane vesicles containing MRP5 was assessed by measuring the uptake of CDCF. The CDCF uptake was measured at 1, 5, 15, and 30 min (Figure 6A). The results revealed a significant time-dependent increase in the CDCF uptake in MRP5 in membrane vesicles after 5 (367%), 15 (137.5%), and 30 min (1817%) compared to the control group (*p* < 0.01 for each). Also, Figure 6B shows that the addition of GSH-Hg-GSH did not alter the uptake of CDCF after 5 min of incubation. The results showed an increase in the CDCF (3170%) and CDCF + GSH-Hg-GSH uptake (2753%) compared with the control group (*p* < 0.05).

**Figure 7:**
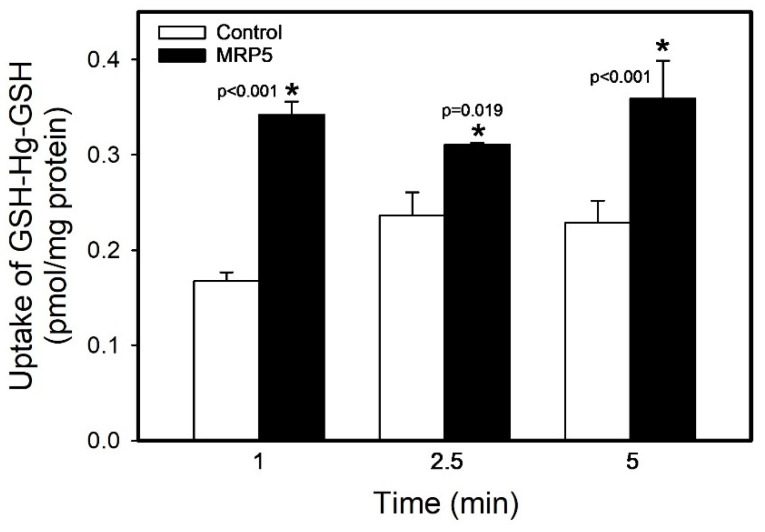
The uptake of GSH-Hg-GSH by MRP5 over time. The results were analyzed using a two-way ANOVA, followed by Tukey’s post hoc test, and are presented as the mean ± the standard error, n = 3. * Significantly different (*p* < 0.05) from the corresponding group of control vesicles.

The transport of GSH-Hg-GSH by MRP5 was assessed over time (at 1, 2.5, and 5 min; Figure 7). The uptake of GSH-Hg-GSH into MRP5 vesicles was significantly greater (104%, 31.2%, and 56.8%, respectively) than that into control vesicles after each time point (*p* < 0.05). Interestingly, the uptake of GSH-Hg-GSH did not increase significantly over time.

**Figure 8:**
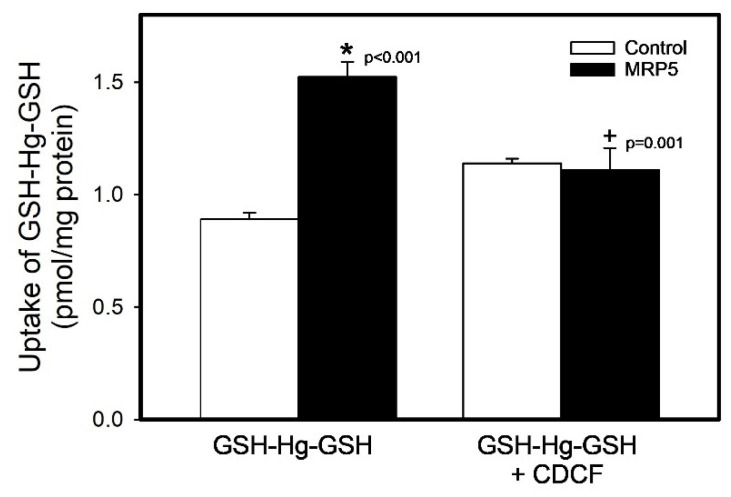
The GSH-Hg-GSH uptake by MRP5 in the presence or absence of CDCF. The results were analyzed using a two-way ANOVA, followed by Tukey’s post hoc test, and are presented as the mean ± the standard error, n = 3. * Significantly different (*p* < 0.05) from the corresponding control group. + Significantly different (*p* < 0.05) from the MRP5 vesicles exposed to GSH-Hg-GSH only.

The uptake of GSH-[^203^Hg]-GSH in the presence and absence of CDCF is shown in Figure 8. The uptake of GSH-[^203^Hg]-GSH into MRP5 vesicles was significantly greater (70.3%) than that into control vesicles (*p* < 0.05). When the GSH-[^203^Hg]-GSH uptake was measured in the presence of CDCF, the uptake was significantly reduced (27.25%) compared with the MRP5 vesicles in the absence of CDCF (*p* < 0.05). Indeed, there was no difference in the uptake of GSH-Hg-GSH between control and MRP5 vesicles in the presence of CDCF.

**Figure 9:**
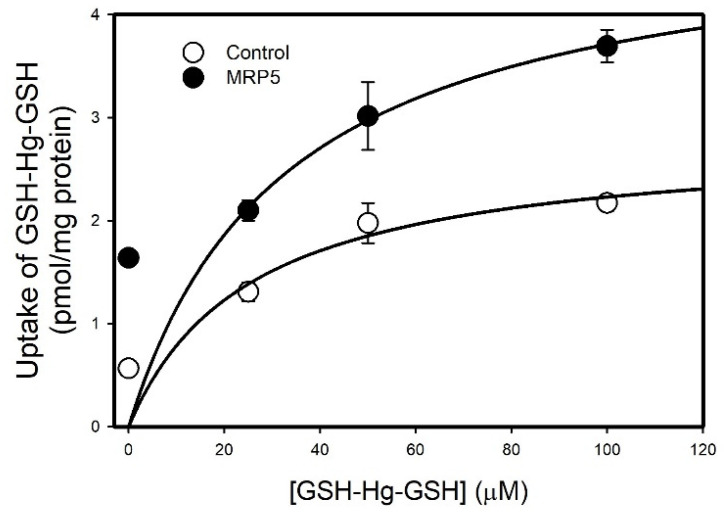
Michaelis–Menten kinetics of GSH-Hg-GSH transport by MRP5 were assessed in inside-out membrane vesicles. Results are presented as mean ± standard error, n = 3.

The Michaelis–Menten kinetics of GSH-Hg-GSH transport by MRP5 were assessed in inside-out membrane vesicles (Figure 9). The uptake of GSH-Hg-GSH was greater in MRP5 vesicles than in the corresponding controls. The *V_max_* for GSH-Hg-GSH transport in the control vesicles was estimated to be 25.58 pmol mg protein^−1^ min^−1^, while the *K_m_* was calculated to be 2.8 μM. For the MRP5 vesicles, the *V_max_* and *K_m_* were estimated to be 32.98 pmol mg protein^−1^ min^−1^ and 4.9 μM, respectively.

## 3. Discussion

The urinary excretion of mercuric ions is an important route of elimination following exposure to all forms of Hg. Therefore, it is critical to understand the processes by which mercuric ions are delivered to the kidney for excretion. GSH-Hg-GSH is thought to be the primary species of Hg filtered at the glomerulus and present in the glomerular filtrate [26]. However, the majority of Hg in the blood appears to be bound to albumin [15], due to the fact that albumin is the most prevalent thiol-containing protein in the plasma. The step by which mercuric ions are removed from albumin and transferred to GSH is unclear. Previous studies have suggested that the liver plays a major role in the processing of mercuric ions before they are delivered to the kidney for excretion [17,18]. In fact, Oliveira et al. [27] demonstrated that the Hg burden increases in the kidney while decreasing in the liver of Wistar rats in a time-dependent manner.

Indeed, findings from in vivo studies in Wistar rats suggest that Hg–albumin conjugates are taken up into hepatocytes through endocytosis and processed intracellularly to form GSH-Hg-GSH [17]. It was postulated that the newly formed GSH-Hg-GSH conjugates are exported into the blood at the sinusoidal membrane for subsequent glomerular filtration and excretion. Two possible candidates for this export are MRP3 and MRP5. These transporters are capable of transporting a wide variety of metabolic waste and xenobiotics [21,28]. Therefore, the purpose of the current study was to investigate the roles of MRP3 and MRP5 in the export of GSH-Hg-GSH conjugates from within hepatocytes.

The present study utilized inside-out membrane vesicles created from HEK cells stably transfected with human MRP3 or MRP5. The vesicular transport system has been used extensively to study the activity of these transporters [21,29]. We confirmed the functionality of these vesicles by measuring the uptake of estradiol or CDCF, which are known substrates of MRP3 or MRP5, respectively [21,22,30]. Our results showed that the uptake of estradiol and CDCF was significantly greater in MRP3 vesicles than in control vesicles, indicating that these membrane vesicles are appropriate models for studies of MRP3 transport. Similarly, the uptake of CDCF was significantly greater in MRP5 vesicles than in controls, indicating that these vesicles are functional models of MRP5 activity.

The ability of MRP3 to mediate the transport of GSH-Hg-GSH was measured directly and indirectly. The uptake of [^3^H]-estradiol into MRP3 vesicles was inhibited significantly by the presence of unlabeled GSH-Hg-GSH. Likewise, the uptake of GSH-[^203^Hg]-GSH was inhibited by the presence of unlabeled estradiol. Based on these findings, we suggest that GSH-Hg-GSH competes with estradiol at the site of MRP3, suggesting that this mercuric conjugate is a substrate of MRP3. To further characterize the transport of GSH-Hg-GSH by MRP3, the uptake of this conjugate was measured over time. The uptake of GSH-[^203^Hg]-GSH into MRP3 vesicles was significantly greater than that into control vesicles. The maximum uptake was achieved between 2.5 and 5 min, which is typical of membrane vesicle experiments. The analysis of saturation kinetics for the uptake of GSH-Hg-GSH also found that the uptake of this conjugate was greater in MRP3 vesicles. Interestingly, previous studies using indirect analyses and modeled predictions of MRP3-mediated GSH transport suggested that GSH and GSH conjugates may not be transported well by this carrier [31,32]. However, the current study provides strong evidence that MRP3 is capable of transporting GSH conjugates, specifically GSH-Hg-GSH. Interestingly, Xu et al. [33] observed that oral exposure to HgCl_2_ and MeHg led to the increased hepatic expression of MRP genes, including MRP3. This finding supports our hypothesis that MRP3 plays a role in the hepatic detoxification of mercuric ions.

We hypothesized previously that MRP5 may also be involved in the export of GSH-Hg-GSH conjugates. The validity of the MRP5 membrane vesicles was confirmed by measuring the uptake of CDCF, a known substrate of MRP5 [30]. The maximum uptake was reached after 5 min. As expected, the addition of unlabeled GSH-Hg-GSH significantly inhibited the uptake of CDCF.

To characterize the transport of GSH-Hg-GSH by MRP5, we analyzed the time course and saturation kinetics of GSH-[^203^Hg]-GSH transport. The uptake was significantly greater in MRP5 vesicles than in the control set at each time studied. Interestingly, the uptake of GSH-Hg-GSH reached the maximum capacity after 5 min, which was similar to the uptake after 1 min. The uptake of GSH-Hg-GSH after 2.5 and 5 min was not significantly different from that at 1 min, suggesting that MRP5-mediated transport is rapid and saturable. The analysis of Michalis–Menten kinetics further confirmed the conclusion that the uptake of GSH-Hg-GSH is due to it being a substrate of this transporter. When radiolabeled GSH-Hg-GSH was incubated with CDCF, the Hg uptake decreased to the levels observed in control vesicles. This suggests that MRP5 transports GSH-Hg-GSH conjugates but has a stronger preference for endogenous substrates.

**Figure 10:**
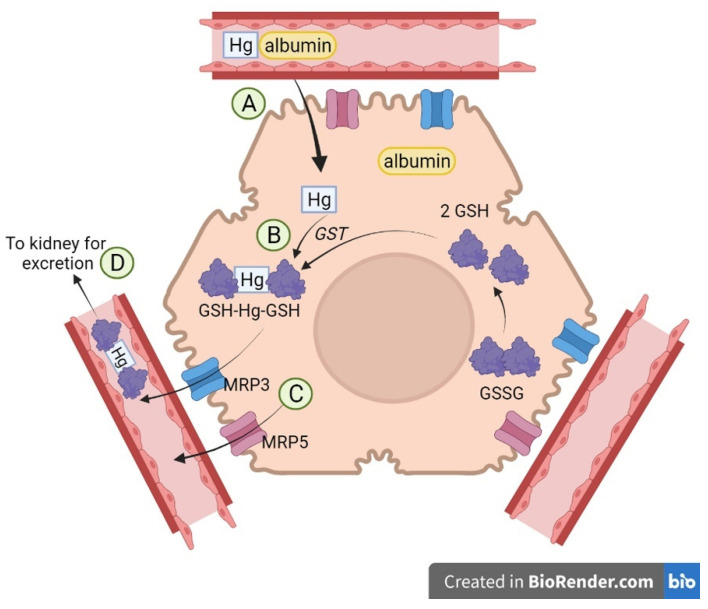
A summary of Hg handling by hepatocytes. Hg–albumin complexes in the blood are taken up through endocytosis at the sinusoidal membrane of hepatocytes (**A**). These complexes are then processed in the cytoplasm to form GSH-Hg-GSH (**B**), which is then transported back into the blood by MRP3 and MRP5 (**C**). These conjugates may then be delivered to the kidney for excretion in the urine (**D**).

In summary, the current data demonstrate that GSH-Hg-GSH is a transportable substrate of MRP3 and MRP5 and that these transporters may play a role in the export of mercuric conjugates from within hepatocytes. These findings support the hypothesis that Hg–albumin conjugates are taken up from the blood into hepatocytes, processed to form GSH-Hg-GSH, and subsequently transported back into the blood via MRP3 and MRP5 for eventual urinary excretion (Figure 10). The current study is limited in that it focused on a single conjugate, GSH-Hg-GSH. Even though this conjugate is relevant, it may not be the only species of Hg that is taken up and processed by the liver. Therefore, while the current study is the first to characterize the roles of MRP3 and MRP5, it is limited by the fact that the studies were carried out in a restricted in vitro environment.

## 4. Materials and Methods

### 4.1. Radioactive Mercury ([^203^Hg])

[^203^Hg] (6–12 Ci mg^−1^) was generated according to a method described previously [34,35]. In summary, 3 mg of mercuric oxide (87% enriched with ^202^Hg) was irradiated for four weeks in the University of Missouri Research Reactor (MURR). After irradiation, the mercuric oxide was dissolved in 1 N trace metal hydrochloric acid and stored at −20 °C until use.

### 4.2. Membrane Vesicles

Inside-out membrane vesicles made from HEK-293 cells transfected with MRP3 (cat no. SBVT15) or MRP5 (cat no. SBVT08) were purchased from Solvo Biotechnology (Budapest, Hungary; distributed by Millipore Sigma, St. Louis, MO, USA). Similarly, control vesicles (cat. no. SBCT06) made from HEK-293 cells transfected with an empty vector were also purchased from Solvo Biotechnology. Vesicular transport assays were carried out according to a previously published protocol [36].

#### 4.2.1. Time Course Analyses of [^3^H]-Estradiol or 5-6-Carboxy-2′,7′-dichlorofluorescein (CDCF) Uptake

The uptake of [^3^H]-estradiol (100 nM) was measured in control and MRP3 vesicles (7.5 µg protein) as a positive control. The vesicles were gently thawed and resuspended using a 25-guage needle. An aliquot of the vesicle suspension (7.5 µg protein) was added to a warm incubation buffer (250 mM sucrose, 10 mM TRIS-HCl, pH 7.4, supplemented with 10 mM MgCl_2_, 10 mM creatine phosphate, 4 mM ATP, and 100 µM.mL-1 creatine phosphokinase) and incubated for 1, 5, 15, or 30 min at 37 °C in a shaking water bath with gentle agitation. In some experiments, the unlabeled conjugate, GSH-Hg-GSH (500 µM), was added to the incubation mixture. GSH-Hg-GSH conjugates were formed by mixing HgCl_2_ with GSH in a 1:2.25 ratio in an incubation buffer. The final concentration of the GSH-Hg-GSH was 100 µM. Following incubation, vesicles were washed in an ice-cold buffer containing 1 mM 2,3-dimercapto propane-2-sulfonate (DMPS) to stop the uptake and wash off any Hg bound to the outside of the vesicles. Each sample was filtered through a MultiScreen plate (0.45 µm; Milipore, Billarica, MA, USA). Filters were removed and the radioactivity contained in each filter was determined using liquid scintillation spectroscopy.

The uptake of 5-6-carboxy-2′,7′-dichlorofluorescein (CDCF) (100 µM) was measured in control and MRP5 vesicles as a positive control. Similarly to the protocol above, the uptake was measured at 37 °C for 1, 5, 15 and 30 min in a shaking water bath with gentle agitation. In some experiments, the uptake of CDCF was measured in the presence of GSH-Hg-GSH (100 µM). The uptake of CDCF into the vesicles was measured using a BioTek^®^ (Winooski, VT, USA) Microplate Reader spectrophotometer at 485 nm (excitation) and 538 nm (emission). 

#### 4.2.2. Uptake of GSH-[^203^Hg]-GSH into MRP3 and MRP5 Membrane Vesicles

MRP3, MRP5, and control vesicles were incubated with 10 µM GSH-[^203^Hg]-GSH for 1, 2.5, or 5 min as described above. To ensure the specificity of the MRP3 uptake, additional experiments were performed wherein 20 µM unlabeled LTC_4_ was added to the GSH-[^203^Hg]-GSH solution for 5 min at 37 °C. MRP5 and control vesicles were incubated with 10 µM GSH-[^203^Hg]-GSH in the presence of the incubation buffer or 1 mM CDCF for 5 min at 37 °C. Following the incubation, vesicles were washed with DMPS and filtered through a MultiScreen plate as described above [36,37].

#### 4.2.3. Analysis of Saturation Kinetics

For the studies of saturation kinetics, vesicles were incubated in 0, 25, 50 or 100 µM unlabeled GSH-Hg-GSH with 10 µM GSH-[^203^Hg]-GSH for 5 min at 37 °C. Following the incubation, vesicles were washed and filtered as described above. Michaelis–Menten kinetics were calculated using Sigma Plot Stat Analysis (Palo Alto, CA, USA).

### 4.3. Data Analysis

The data were analyzed using the Kolmogorov–Smirnov normality test and Levene’s test for the homogeneity of variances. Subsequently, data were analyzed using a two-way ANOVA followed by Tukey’s post hoc multiple comparison test. The results were considered statistically significant when *p* < 0.05. For the enzyme kinetics analysis, the Michaelis–Menten equation was used in order to estimate the maximum velocity (*V_max_*) and the Michaelis constant (*K_m_*).

## Data Availability

Original data will be provided upon request.

## Supplementary Materials

The following supporting information can be downloaded at: https://www.mdpi.com/article/10.3390/ijms26031194/s1.
