# Supplementary figures and images for: Multidrug Resistance-Associated Proteins 3 and 5 Play a Role in the Hepatic Transport of Mercuric Conjugates of Glutathione
Source: Int J Mol Sci. 2025 Jan 30;26(3):1194. doi: 10.3390/ijms26031194 (PMC11818351; doi:10.3390/ijms26031194)

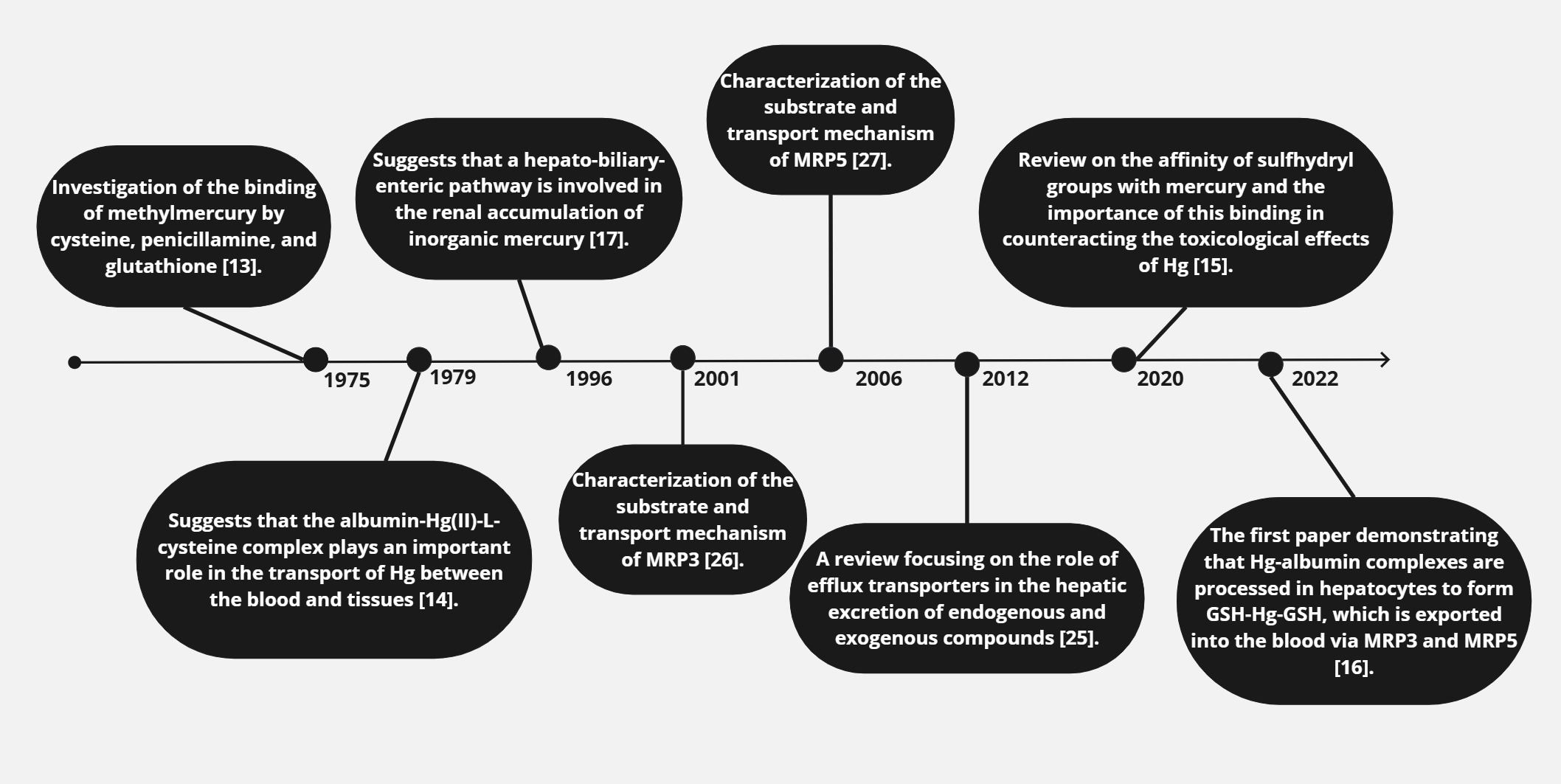

Supplement: Supplementary file 1 [file ijms-26-01194-s001.zip › ijms-3412834-supplementary.jpg]
